# Prevalence and Subtype Distribution of *Blastocystis* sp. in Senegalese School Children

**DOI:** 10.3390/microorganisms8091408

**Published:** 2020-09-12

**Authors:** Salma Khaled, Nausicaa Gantois, Amadou Tidjani Ly, Simon Senghor, Gaël Even, Ellena Dautel, Romane Dejager, Manasi Sawant, Martha Baydoun, Sadia Benamrouz-Vanneste, Magali Chabé, Seynabou Ndiaye, Anne-Marie Schacht, Gabriela Certad, Gilles Riveau, Eric Viscogliosi

**Affiliations:** 1 Institut Pasteur de Lille, U1019–UMR 9017–CIIL–Centre d’Infection et d’Immunité de Lille, University of Lille, CNRS, Inserm, CHU Lille, F-59000 Lille, France; salmakhaled94@outlook.com (S.K.); nausicaa.gantois@pasteur-lille.fr (N.G.); dautel.ellena@gmail.com (E.D.); romanedejager97@gmail.com (R.D.); manasi.sawant@pasteur-lille.fr (M.S.); martha.e.b@hotmail.com (M.B.); sadia.vanneste@gmail.com (S.B.-V.); magali.chabe@univ-lille.fr (M.C.); am.schacht@gmail.com (A.-M.S.); gabriela.certad@pasteur-lille.fr (G.C.); gilles.riveau@gmail.com (G.R.); 2 Biomedical Research Center Espoir Pour La Santé (BRC-EPLS), BP 226 Saint-Louis, Senegal; tidjani.ly@espoir-sante.org (A.T.L.); simon.senghor@espoir-sante.org (S.S.); 3 Gènes Diffusion, F-59501 Douai, France; g.even@genesdiffusion.com; 4 PEGASE-Biosciences (Plateforme d’Expertises Génomiques Appliquées aux Sciences Expérimentales), Institut Pasteur de Lille, F-59000 Lille, France; 5 Laboratoire Ecologie et Biodiversité, Institut Catholique de Lille, Faculté de Gestion Economie et Sciences, F-59000 Lille, France; 6 Région Médicale de Saint-Louis, MSAS, BP 226 Saint-Louis, Senegal; zeynab43@yahoo.fr; 7 Délégation à la Recherche Clinique et à l’Innovation, Groupement des Hôpitaux de l’Institut Catholique de Lille, F-59000 Lille, France

**Keywords:** *Blastocystis* sp., intestinal parasites, Africa, Senegal, molecular epidemiology, real-time quantitative PCR, SSU rDNA sequence, subtyping, transmission, zoonosis

## Abstract

*Blastocystis* sp. is an enteric protozoan that frequently colonizes humans and many animals. Despite impacting on human health, data on the prevalence and subtype (ST) distribution of *Blastocystis* sp. remain sparse in Africa. Accordingly, we performed the first multicenter and largest epidemiological survey ever conducted on *Blastocystis* sp. for this continent. A total of 731 stool samples collected from healthy school children living in 10 villages of the northwestern region of Senegal were tested for the presence of *Blastocystis* sp. by real-time polymerase chain reaction followed by subtyping of positive samples. Considerable variation in prevalence between villages (51.7 to 100%) was evident with the overall prevalence being 80.4%. Mixed infections were identified in 23% of positive individuals. Among 453 school children with a single infection, ST2 was predominant, followed by ST1, ST3, ST7, ST10, and ST14; this is the first report of ST10 and ST14 in humans. Genetic polymorphisms were evident at the intra-ST level with the identification of numerous ST1 to ST3 genotypes. ST1 showed the greatest intra-ST diversity followed by ST2 and ST3. The prevalence and distribution of STs and genotypes varied among target villages, pointing to several potential infection sources, including human-to-human, zoonotic, and waterborne transmission.

## 1. Introduction

*Blastocystis* sp. is an enteric protozoa with a worldwide distribution, frequently found in human as well as in various animal groups spanning from non-human primates to insects [1,2,3,4,5,6]. As the main mode of transmission of this parasite is the fecal–oral route through the host colonization by the transmissible environment-resistant cystic form [7], its prevalence in the human population is strongly correlated to sanitary conditions, hygiene practices, and quality of drinking water. Not surprisingly, the prevalence of *Blastocystis* sp. is thus much lower in industrialized countries than in lower-income geographical areas [5]. For instance, it reaches 10 to 20% in European countries [8,9,10,11] but can largely exceed 40–50% in various African [12,13], Asian [14,15], and American countries [16,17].

The high prevalence of *Blastocystis* sp. described in healthy individuals also raises the long-standing question of its real pathogenicity and clinical impact in the human population [5,18,19,20]. Indeed, this single-celled microorganism was commonly found in individuals with neither any gastrointestinal complaints nor symptoms. In contrast, it was the only causative agent identified in the stools of patients presenting gastrointestinal symptoms such as diarrhea or abdominal pain [21] but also urticaria [22]. Therefore, under certain circumstances or interactions with the host, colonization by *Blastocystis* sp. becomes infection [5,6,21,22]. In that respect, recent in vitro studies have led to the identification of molecules and mechanisms potentially involved in the pathogenicity of *Blastocystis* sp. [23].

An extensive genetic diversity has been highlighted among numerous *Blastocystis* sp. isolates from both humans and animal hosts on the basis of small subunit (SSU) rDNA gene sequences analysis [24,25]. Formerly, 17 separate lineages, so-called subtypes (STs) arguably species, had been clearly recognized among mammalian and avian isolates (ST1 to ST17) [26]. Recently, epidemiological surveys conducted in various mammalian and non-mammalian (reptiles, fish, and insects) sources described isolates representative of potential novel STs [3,4,27,28,29,30]. However, several if not all of these proposed STs are likely invalid and should be rejected at this time until near-complete SSU rDNA gene sequences should be generated.

Among the 17 mammalian and avian STs recognized so far, 10 of them (ST1–ST9 and ST12) have been identified in the human population even if ST1–ST4 appear by far to be the most common colonizers of the human intestinal tract [19,31,32]. Indeed, ST1–ST4 represent nearly 95% of the subtyped isolates in the world, which is consistent with large-scale parasite spread directly from person to person (anthroponotic transmission). Conversely, the rarer STs in humans (ST5–ST9 and ST12) are frequently harbored by various animal groups including non-human primates (ST8) [4,33], hoofed animals (ST5) [34], and birds (ST6 and ST7) [35], strongly suggesting the zoonotic origin of these STs in humans.

Despite the current burden of *Blastocystis* sp. in humans, only sparse molecular studies provide information on the prevalence and ST distribution of *Blastocystis* sp. in the African population. However, most African countries are considered to be at high risk of *Blastocystis* sp. infection due to poor access to sanitation and clean water, thus favoring parasite transmission. To date, the available data mainly concerns few countries of the Northern, Western, and Central Africa such as Libya [31], Egypt [36], Nigeria [37], Côte d’Ivoire [38], Senegal [12], and Cameroon [13]. Moreover, these epidemiological studies remained based on cohorts still too limited in size. Consequently, it is necessary to keep expanding the molecular epidemiology of *Blastocystis* sp. in Africa through additional studies to further clarify aspects of prevalence, ST distribution, and potential sources of transmission of this parasite. Therefore, the aim of the present study was to reinforce the picture of *Blastocystis* sp. impact and circulation by performing the first multicenter and largest epidemiological survey ever conducted in Africa.

## 2. Materials and Methods

### 2.1. Ethics Approval

The present study was sponsored by the Biomedical Research Centre Espoir pour la Santé (Saint-Louis, Senegal: www.espoir-sante.org) and approved by the National Ethics Committee of the Ministry of Health and Social Action of Senegal (reference number 000146/MSAS/DPRS/CNERS; protocol number SEN19/43; Date of approval: 08/27/2019). Written and oral informed consents were obtained from the parents or the legal guardians of the children for biological samples. The subjects’ data were collected anonymously (with encryption of the identity of individuals). This study was conducted in accordance with the Code of Ethics of the World Medical Association (Declaration of Helsinki III) and with the International Ethical Guidelines for Biological Research Involving Human Subjects.

### 2.2. Sampling Sites

**Figure 1:**
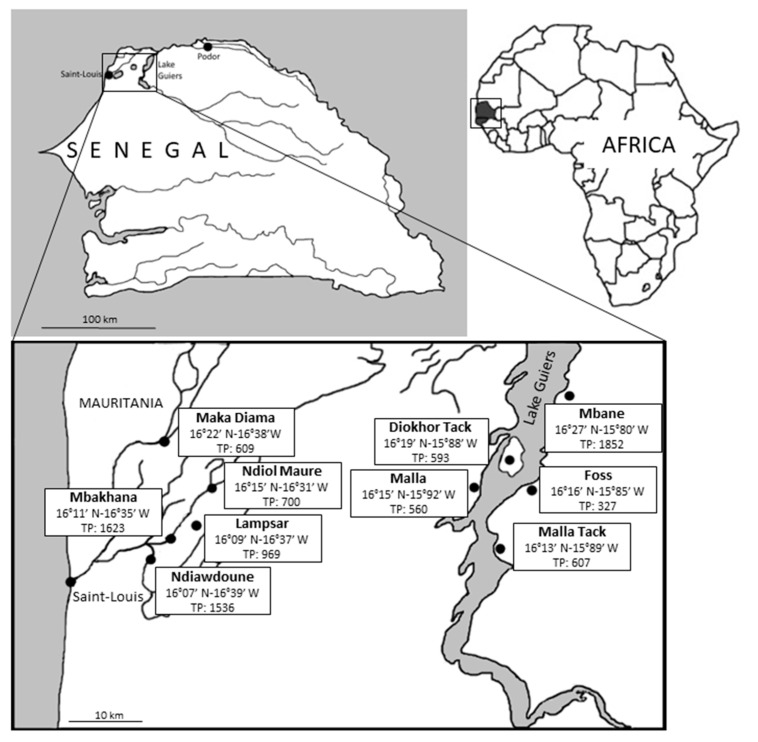
Detailed location of the 10 villages in Northwestern Senegal screened for the presence of *Blastocystis* sp. by qPCR. Geographic coordinates and total population (TP) of each of the villages are indicated.

The study was conducted in 10 villages of the Saint-Louis (Maka Diama, Ndiol Maure, Lampsar, Mbakhana, Ndiawdoune, Mbane, Diokhor Tack, and Malla) and Louga (Foss and Malla Tack) regions located in northwestern Senegal. The geographical coordinates of the villages together with their total population are indicated in Figure 1. Five of these villages are located in the Senegal River Basin (so-called herein area of Saint-Louis), either on the banks of the Senegal River (Maka Diama), Lampsar River (Ndiol Maure, Lampsar and Mbakhana), or Ngalam River (Ndiawdoune). The five other villages are located around the Lake Guiers (so-called area of Lake Guiers). Lake Guiers is mainly supplied to the north by the Senegal River, to which it is connected by the canalized Taouey River and represents a chief source of fresh water for the city of Dakar. The two areas of Saint-Louis and Lake Guiers are separated by ~50 km.

Throughout the villages, the habitat is of urban type with houses built as permanent constructions except for the village of Maka Diama where some traditional dwellings subsist. Regarding sanitary and environmental conditions, all villagers use traditional latrines, isolated pit latrines with (very few) or without flushing or open-air toilet facilities (mainly children). In contrast, water supplies and their use (drinking; washing; bathing; domestic activities such as cooking, household cleaning, dishes, and laundry; and irrigating crops) differ between villages according to their geographical location and socio-economic status (Table 1). With the exception of Maka Diama, Ndiol Maure, Mbakhana, and Mbane, all other villages have a fishing activity which remains rather limited. In addition, the breeding of farm animals (cattle, sheep, goats, donkeys, chickens, and horses) is practiced in all the villages.

### 2.3. Cohort and Collection of Samples

Sampling was conducted between August 2019 and October 2019. During the warm season (March to October), temperature ranges between 25 °C and 45 °C. Under the supervision of school directors, 25 to 104 stool samples were collected in the schools of each of the 10 villages from elementary school children (1 sample per individual). Date of birth of each child was ascertained from vaccination cards or school register. A total of 731 stool samples were thus collected from boys (*n* = 379) and girls (*n* = 352) (sex ratio M/F of 1.08) and the age of participants was between 6 and 19 years (mean age of 10.5 ± 2.09 years). Sampling size was quite similar between the two study areas of Saint-Louis (363 stool samples) and Lake Guiers (368). For each school child, ~2 g of fresh stool was added to 2 mL of 2.5% potassium dichromate (*w/v* in water) (Sigma Life Sciences, Saint-Louis, MO, USA) in a sterile tube then homogenized by shaking. All samples were transported at 4 °C to the Institut Pasteur of Lille (France).

### 2.4. DNA Extraction and Molecular Subtyping of Blastocystis sp. Isolates

One mL of diluted stool from each sample was stirred and then centrifuged 3 times for 10 min at 3000× *g* with water to remove all traces of potassium dichromate. The resulting stool pellet was diluted in 500 µL of water. Total genomic DNA was extracted from the diluted pellet using the QIAamp DNA Stool Mini Kit (Qiagen GmbH, Hilden, Germany) according to the manufacturer’s recommended procedures. DNA extraction negative controls were included. DNA was eluted in 100 µL of elution (AE) buffer provided in the DNA extraction kit and stored at −20°C until being analyzed. For each sample tested, 2 µL of extracted DNA was subjected to a qPCR assay using the Blastocystis-specific primers BL18SPPF1 (5′-AGTAGTCATACGCTCGTCTCAAA-3′) and BL18SR2PP (5′-TCTTCGTTACCCGTTACTGC-3′) targeting the small subunit (SSU) rDNA gene [39]. DNA extraction controls were also used in qPCR assays and both positive (DNAs from *Blastocystis* sp. ST7 and ST4) and negative (DNA matrix replaced by water) qPCR controls were included. The positive qPCR products were purified and directly sequenced on both strands by Genoscreen (Lille, France). For a significant proportion of samples, sequence chromatograms analysis revealed the presence of double traces, suggesting infections by at least two different *Blastocystis* STs. In these cases, the STs present were not determined and these positive samples were considered as mixed infections. The SSU rDNA sequences obtained in this study were deposited in GenBank under accession numbers MT621678 to MT622130. These sequences were compared with all *Blastocystis* sp. homologous sequences of known STs available from the National Centre for Biotechnology Information (NCBI) using the nucleotide Basic Local Alignment Search Tool (BLAST) program. The STs were identified by determining the exact match or closest similarity against all known mammalian and avian *Blastocystis* sp. STs according to the most recent classifications of the parasite [4,26]. Subsequently, the sequences of *Blastocystis* sp. isolates belonging to the same ST (ST1, ST2 or ST3) were aligned with each other using the BioEdit v. 7.0.1 package (Date of release 06/10/2019) (http://www.mbio.ncsu.edu/BioEdit/bioedit.html) to determine intra-ST diversity and identify so-called genotypes referring to genetically distinct strains within a same ST. The term “genotype” was proposed in a previous survey [35] to prevent any confusion with the term “allele” already used by others and designed through the comparison of sequences from another domain of the SSU rDNA gene [18,40].

### 2.5. Statistical Analysis

For the statistical analysis, Fisher’s exact test was used to test the relationship between different categorical variables. Logistic regression models were created to calculate odds ratios (OR) and 95% confidence interval (CI) considering *Blastocystis* sp. colonization, STs, and genotypes as the main outcomes. The general significance level was set at a *p*-value below 0.05. All analyses were performed using packages stats and OR from the R statistical computing program v. 3.6.1 (Date of release 07/05/2019) (R Development Core Team; http://www.R—project.org).

## 3. Results

### Prevalence of Blastocystis sp. in the Cohort of Senegalese School Children

Single stool samples were collected from a total of 731 school children living in 10 villages of the region of Saint-Louis in Senegal. All were considered asymptomatic due to the apparent absence of digestive disorders. The overall prevalence of *Blastocystis* sp.-positive individuals was shown to be 80.4% (588/731) using qPCR (Table 2). The difference in prevalence observed between males (51.5%) and females (48.5%) colonized by the parasite was not significant (OR: 0.937, CI: 0.649–1.351, *p* = 0.729). In addition, the mean age of *Blastocystis* sp.-colonized children was not significantly different compared to the age of *Blastocystis* sp.-free subjects (mean age of 10.54 ± 2.12 years versus 10.36 ± 1.99; OR: 1,28, CI: 0.755–2.219, *p* = 0.3684). The prevalence of the parasite was then analyzed separately in the two areas of Saint-Louis and Lake Guiers. The average prevalence observed in the area of Saint-Louis was slightly significantly higher than that of the area of Lake Guiers (83.5% versus 77.4%; OR: 1.471, CI: 1.018–2.134, *p* = 0.04). At the village level, the prevalence of *Blastocystis* sp. was highly variable, ranging from 51.7% to 100% (Table 2). Within the area of Saint-Louis, the parasite was significantly more prevalent in the village of Ndiawdoune than in the 4 other villages of the same area (OR: 11.58043, CI: 1.899–474.992, *p* = 0.0009), while it was significantly less prevalent in the village of Ndiol Maure (OR: 0.277, CI: 0.145–0.531, *p* < 0.0001). In the same way, within the area of Lake Guiers, the prevalence of *Blastocystis* sp. was significantly higher in the villages of Malla (OR: 5.794, CI: 11.794–29.83, *p* = 0.000643) and Malla Tack (OR: 2.775, CI: 1.374–6.103, *p* = 0.003) and lower in the one of Mbane (OR: 0.180, CI: 0.102–0.317, *p* < 0.0001).

The qPCR products of the 588 samples positive for *Blastocystis* sp. were all sequenced on both strands. For 135 of these samples (23%), sequence chromatogram analysis revealed the presence of double traces, reflecting mixed infection by different STs that were not identified. The remaining 453 isolates corresponded to single infection by one ST. All partial SSU rDNA gene sequences obtained from these 453 positive samples showed 98 to 100% identity with homologous sequences available in databases for known STs (Table 2). ST2 was predominant (*n* = 226, 49.9%), followed by ST1 (*n* = 113, 24.9%), ST3 (*n* = 107, 23.6%), ST7 (*n* = 3, 0.7%), ST10, and ST14 (both *n* = 2, 0.45%). This distribution of *Blastocystis* sp. STs in our overall population was not significantly associated with gender (Fisher’s exact test, *p* = 0.3) or age (Fisher’s exact test, *p* = 0.75) of the individuals. On the other hand, significant difference in the distribution of predominant STs (ST1 to ST3) was found between the area of Saint-Louis and that of the Lake Guiers (Fisher’s exact test, *p* = 0.0007). A strong overabundance of ST3 was identified in the area of Saint-Louis (OR: 2.398, CI: 1.529–3.819, *p* = 0.0002), whereas ST2 was slightly more frequently found in the area of Lake Guiers (OR: 0.679, CI: 0.467–0.985, *p* = 0.04). The distribution of STs also varied between the villages, even within the same area and even though ST2 was predominant in 8 of the 10 villages screened. ST1 was significantly less abundant in Lampsar (OR: 0.39, CI: 0.155–0.862, *p* = 0.01), Malla (OR: 0.217, CI: 0.042–0.706, *p* = 0.004) and Ndiawdoune (OR: 0, CI: 0–0.308, *p* < 0.0001), and more reported in Ndiol Maure (OR: 2.778, CI: 1.335–5.731, *p* = 0.03) and Mbane (OR: 2.597, CI: 1.233–5.404, *p* = 0.01), while ST3 was more predominant in Lampsar (OR: 2.935, CI: 1.606–5.320, *p* = 0.003) and less frequently reported in Malla (OR: 0.236, CI: 0.046–0.772, *p* = 0.01) and Diokhor Tack (OR: 0.371, CI: 0.138–0.855, *p* = 0.01). Moreover, ST2 was significantly more frequently found in Malla (OR: 6.322, CI: 2.55–18.83, *p* < 0.0001), Ndiawdoune (OR: 2.50, CI: 1.112–6.015, *p* = 0.02) and less reported in Mbane (OR: 0.368, CI: 0.160–0.791, *p* = 0.006), Maka Diama (OR: 0.490, CI: 0.236–0.984, *p* = 0.04), and Ndiol Maure (OR: 0.403, CI: 0.181–0.851, *p* = 0.01). Rare human STs including ST7, ST10, and ST14 were more frequently identified in the area of Saint-Louis (identification of three ST7, one ST10, and two ST14 isolates) than in the area of Lake Guiers (identification of a single ST10 isolate).

**Figure 2:**
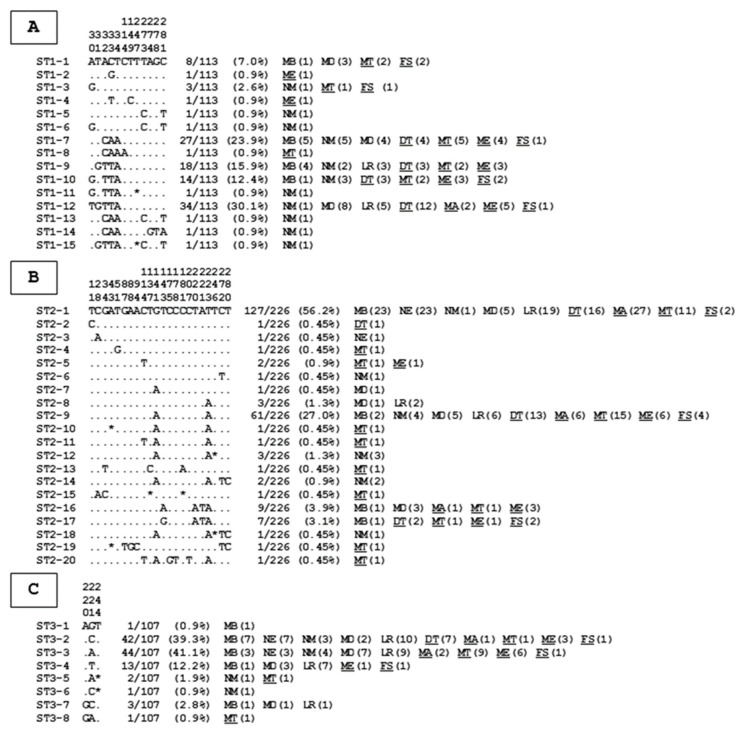
Alignment of partial SSU rDNA gene sequences from *Blastocystis* sp. ST1 (**A**), ST2 (**B**), and ST3 (**C**) isolates. Only the variable positions identified in the compared domain of the gene for these STs are shown in this alignment. The positions of variable nucleotides with respect to the reference sequences (genotypes ST1-1, ST2-1, and ST3-1) are indicated above the alignment (vertical numbering). Nucleotides identical to those of the reference sequences are represented by dashes and gaps are represented by asterisks. All the genotypes identified for each ST are indicated on the left of the alignment. On the right of the alignment is reported the total number and percentage of isolates identified in our study for each genotype followed by their repartition per village (number of isolates in parentheses). Abbreviations: MB, Mbakhana; MD, Maka Diama; NM, Ndiol Maure; LR, Lampsar; NE, Ndiawdoune; MT, Malla Tack; FS, Foss; ME, Mbane; DT, Diokhor Tack; MA, Malla. The abbreviations of the villages located in the area of Lake Guiers are underlined, which is not the case of those located in the area of Saint-Louis.

All the partial SSU rDNA gene sequences representative of the same ST were aligned with each other to assess intra-ST diversity. The 113 ST1 sequences showed 97.2% to 100% identity between them. By comparing all these ST1 sequences, 12 positions were reported as variable, i.e., positions exhibiting at least one nucleotide difference within at least one of the compared sequences (Figure 2), allowing the identification of 15 so-called genotypes (ST1-1–ST1-15).

The ratio between the number of ST1 isolates and the number of ST1 genotypes was 7.5 (113/15). Among these genotypes, four of them (ST1-7, ST1-9, ST1-10, and ST1-12) identified in six to seven villages comprised more than 80% of the ST1 isolates (93/113), while nine other ST1 genotypes were only represented by a single isolate. Regarding ST2 sequences, their identity with each other ranged from 95.8 to 100%. The alignment of the 226 ST2 sequences allowed the identification of 22 variable positions, leading to 20 ST2 genotypes (ST2-1 to ST2-20), with an average of 11.3 isolates per genotype (226/20). Only two of these genotypes, ST2-1 and ST2-9, both present in nine of the 10 villages screened, accounted for more than 80% of the ST2 isolates (188/226). In contrast, more than half of the ST2 genotypes (12/20) were represented by a single isolate. The identity between the sequences of the 107 ST3 isolates was comprised between 98.9 and 100%, with the identification of only three variable positions, leading to 8 ST3 genotypes (ST3-1 to ST3-8). The ratio between the number of isolates per ST3 genotype reached 13.4 (107/8). The two predominant genotypes, ST3-2 and ST3-3, included more than 80% of the ST3 isolates and were found in 10 and nine villages, respectively. On the other hand, three ST3 genotypes were represented by only one isolate (ST3-1, ST3-6, and ST3-8).

In the two compared areas, the number of ST1 to ST3 genotypes identified was roughly similar (30 genotypes in the area of Saint-Louis versus 27 in the area of Lake Guiers). However, genotypes ST3-2 (OR: 2.311, CI: 1.184–4.741, *p* = 0.02) and ST3-4 (OR: 5.47, CI: 1.443–35.67, *p* = 0.03) were more frequently reported in the area of Saint-Louis, while ST2-9 was predominantly found in the area of Lake Guiers (OR: 0.306, CI: 0.164–0.549, *p* = 0.0001). A large variability in the number of genotypes was also reported between villages even within the same region (Table 3). Within the area of Saint-Louis, the calculated ratio between the total number of subtyped isolates (ST1, ST2, and ST3) and the total number of genotypes identified from each village ranged between 1.9 and 8.5), for an area average of 7.6 isolates per genotype. In the area of Lake Guiers, this ratio showed a slightly narrower range, between 1.6 and 6.8. The average ratio within this area (8.0) was quite similar to that calculated for the area of Saint-Louis. Moreover, ST3-4 and ST2-9 were the only genotypes that were significantly more abundant in our survey, respectively, in Lampsar (OR: 7.0, CI: 1.93–26.34, *p* = 0.0007) and Malla Tack (OR: 3.13, CI: 1.45–6.56, *p* = 0.001).

Regarding rarer STs such as ST7, sequences of three isolates were compared, showing 99.6 to 100% identity (only one variable position). In case of ST10 and ST14, two sequences were obtained and compared for each of these two STs, exhibiting 98.9 (3 variable positions) and 96.9% (9 nucleotide differences) identity, respectively.

## 4. Discussion

The present study represents the largest-scale epidemiological survey ever conducted in Africa regarding the prevalence and ST distribution of *Blastocystis* sp. and gives a more comprehensive overview of the parasite circulation in African developing countries. The overall prevalence observed herein among Senegalese school children reached 80.4%, highlighting the burden of *Blastocystis* sp. in this part of the world. This is in line with a previous study also conducted in Senegal in which the prevalence of *Blastocystis* sp. reached 100% among a limited cohort of ~100 children living in the Podor district located further north of the country [12]. This represented the highest prevalence of the parasite ever observed worldwide. Strikingly, a prevalence of 100% was also reported in the present study in the village of Ndiawdoune by screening a limited cohort of 50 school children (Table 2).

The data obtained herein were thus compared with those reported in other African regions and countries (Table 4). In this aim, only data obtained by molecular methods were recorded as non-molecular methods such as microscopic observation of fresh feces are known to greatly underestimate the prevalence of the parasite [39,41]. The prevalence of the parasite ranges between 25.6% and 100% depending on the geographical area [12,13,31,36,37,38,41,42,43,44,45,46,47,48,49,50,51,52,53]. By focusing on the neighboring countries of Senegal (West Africa), the prevalence of *Blastocystis* sp. was, respectively, 58.2% [38] and 70% [45] in two different surveys performed in Côte d’Ivoire and 49.7% in Mali [46]. Two other field studies showed a prevalence of 49% [31] and 84% [37] in Nigeria, while only one small-scale cohort screened in Liberia [31] indicated a frequency of the parasite of ~70%. All these epidemiological data point to the high prevalence of the parasite in Africa, with an average of over 50% in numerous countries. It is thus clear that significant prevalence values in African developing countries are associated with fecal peril in link with precarious sanitary conditions and quality of drinking water. Moreover, promiscuity in schools is an additional factor that could likely facilitate the transmission of the parasite.

At the regional level, the prevalence of *Blastocystis* sp. was of the same order of importance between the two Senegalese study areas of Saint-Louis (83.5%) and Lake Guiers (77.4%). Nevertheless, a large variation was reported between villages of these two areas that could scarcely be explained by differences in sanitary conditions encountered in the villages. Indeed, the habitat is of urban type in all the villages and all inhabitants use latrines. In contrast, various origins of water sources for drinking, domestic activities, and irrigated crops potentially contaminated with human and animal feces might account for the observed variation of the waterborne parasite frequency. For instance, the highest prevalence of *Blastocystis* sp. has been reported in the village of Ndiawdoune. Within the area of Saint-Louis, this is the only village to be located on the banks of the Ngalam River (Table 1), which might exhibit a potential higher level of contamination by the parasite than the Senegal River or the Lampsar River. Regarding the villages of the area of Lake Guiers, the water sources tended to be more diversified than for the area of Saint-Louis (Table 1), even though the lake water represents the main one. Interestingly, the prevalence of *Blastocystis* sp. gradually increased in villages located from north to south of the Lake Guiers. Indeed, Mbane, the northernmost village, exhibited a prevalence of 51.7% followed to the south by Diokhor Tack (76%) and then further south by the villages of Foss, Malla, and Malla Tack with a prevalence reaching or exceeding 90%. Interestingly, Mbane is the closest to the geographical zone in which the lake is supplied by the Taouey River. In this zone, stagnation of water together with fecal debris could likely be reduced, leading to a more limited contamination of the villagers. In addition, Mbane is the only village of this area with tap water unlike the inhabitants of other villages in the same area whose main water supply is the lake. Moreover, lake water flowing to villages further north within a closed aquatic system could stagnate and accumulate fecal debris from villages located further south and thus potentially increase the risk of infection by *Blastocystis* sp. This stagnation in the vicinity of the water points of these villages is also important because of the extreme density of aquatic vegetation on the shores of the lake. These hypotheses remain to be further confirmed through the search for the parasite in water samples collected near the Senegalese villages.

Within our overall Senegalese cohort, ST2 was largely predominant (49.9% of the subtyped isolates) followed by ST1 (24.9%) and ST3 (23.6%). Additional STs including ST7 (0.7%), ST10, and ST14 (both 0.45%) were poorly represented. This ST distribution could first be compared with that observed in a previous survey conducted in children of the Podor district in Senegal, reporting the predominance of ST3 followed by ST1 and ST2 [12]. In other West African countries like Nigeria [31,37] and Côte d’Ivoire [38], ST1 was the predominant variant followed by either ST2 or ST3. Variation in ST distribution was also observed between other African regions as ST3 was predominant in Central [13,42] and North [36,47,49,50,51] Africa, with the exception of Libya, where ST1 was the most frequent ST [31,48] as in Southern Africa [44]. Although all these data were often based on a limited number of individuals, a geographical variation in the frequency of the three major STs was reported between African regions or within the same country that might simply reflect different exposure to environmental and/or animal infection sources. This variation was also described within the same region as shown in the present survey in the zone of Saint-Louis. Indeed, ST3 was the most commonly detected ST in the area of Saint-Louis while it was ST2 in the area of Lake Guiers. Still, in West Africa, but in Nigeria, ST2 was the second most identified ST after ST1 in a community living in the town of Ilero (Oyo state) [37], while ST2 was surprisingly not detected in samples collected in patients attending a clinic in Lagos (Lagos state) [31].

Overall, ST1, ST2, and ST3 isolates represent the huge majority of human isolates identified in Senegal and more generally in Africa with 95.1% (1550/1630) of the total of isolates subtyped until now on this continent, together with an overall predominance of ST3. As these three STs are frequently found in the human population and globally more rarely in animals [4], it can be concluded that ST1 to ST3 infections in humans are mostly the result of large-scale human-to-human transmission, which can be reasonably enhanced in low-income countries such as Senegal in which an increase in the number of cases of waterborne illnesses of humans and animals living in the Senegal River Basin has been reported in the last years [54].

Interestingly, ST4 has not been identified in the present epidemiological survey and a total of only nine ST4 isolates have been characterized in Africa out of the 1630 isolates subtyped so far on this continent (0.55%) (Table 3). In contrast, ST4 appears to be common, primarily in Europe [8,31,39]. Our observations therefore support the hypothesis that ST4 entered the human population in the recent past in comparison to others STs [18]. However, the potential zoonotic source of this ST4 emergence likely in the European population still remains unknown.

In Senegal, three cases of ST7 infection were identified in our study, all of them in the area of Saint-Louis. It has been confirmed that ST6 and ST7 represent avian-adapted STs in view of their large predominance in birds [35]. In Africa, these two STs together represent only ~4% (66/1630) of the human isolates subtyped (Table 3), confirming that “avian STs” are rare in humans outside of Asia [18,19]. The most likely origin of these ST7 isolates is zoonotic from farm poultry but the mode of transmission between hosts is not conclusively demonstrated. Indeed, it can occur either through intimate contact of humans with animals (fecal–oral route) or through consumption of water or food contaminated with bird feces. To complete this overview, ST10 and ST14 were identified in four school children living in different villages, indicating independent sources of contamination. Numerous studies have reported that ST10 and ST14 were largely predominant in bovid, confirming that both STs could be considered as adapted to this animal group [55,56]. However, despite the high prevalence of these two STs in bovid and potential long-term and repeated contact with these animals, especially for farmers, ST10 and ST14 had never been documented in human infections worldwide prior to this survey [18,19]. As for ST7, the mode of transmission of ST10 and ST14 to humans remains speculative and may be through either direct contact with livestock or waterborne transmission, as ST10 has already been detected in river water samples collected for instance in Peninsular Malaysia [57].

Due to the large number of isolates subtyped herein, the intra-ST diversity of the three major STs identified in our Senegalese cohort (ST1 to ST3) could be thoroughly investigated. Strikingly, these three STs appeared very different in terms of their intra-ST variation that was evaluated through the ratio between the number of isolates and the number of genotypes from each ST (Table 3). In case of ST1, 15 genotypes were reported with an average of 7.5 isolates per genotype. Regarding ST2, 20 genotypes were registered, thus suggesting a greater intra-ST genetic diversity than ST1. However, the ratio between the number of isolates and the number of ST2 genotypes reached 11.3, rebutting this assumption. Noticeably, ST3 exhibited the lower intra-ST diversity as only eight genotypes were identified with an average of 13.8 isolates per genotype. For comparison, very few studies have previously focused on the intra-ST genetic diversity among these three major STs and almost all were based on the analysis of sequences from another domain of the SSU rDNA gene than the one targeted in our study [18,40]. Through the analysis of this second marker, it was also reported that genetic diversity could vary dramatically at the intra-ST level in epidemiological surveys conducted for instance in China [58] and Nigeria [37]. Indeed, ST2 was shown to exhibit the greater intra-ST diversity (average of 2.2 isolates per genotype) followed by ST1 (6.7) then ST3 (21.5) in the Chinese study. In Nigeria, ST3 was far more divergent (5.6) then ST2 (13) and ST1 (20). Interestingly, intra-ST diversity also largely fluctuated between different geographical regions within the same country as shown from two studies conducted in Colombia. In one of these surveys [59], the greater intra-ST diversity was reported for ST2 (7) followed by ST1 (9.2) and ST3 (15.8), while in the second [17], ST2 was the most divergent ST (4.2) ahead of ST3 (4.7) and ST1 (9.6). Globally, our data coupled with those obtained these previous studies highlighted the lower intra-ST genetic diversity of ST3 compared to ST1 and ST2. Interestingly, this was confirmed through the comparative analysis of a large number of draft *Blastocystis* sp. genomes reconstructed from shotgun metagenomics data [43].

It is known that ST1 and ST2 can colonize different groups of animals while ST3 is mainly found in humans [4]. This highlighted the relatively high host specificity within ST3 as previously proposed [40], strongly suggesting that most ST3 infections in humans are caused predominantly by anthroponotic transmission. This was emphasized in the present study as three of the eight ST3 genotypes identified represented more than 90% of ST3 isolates. Consequently, the remaining five ST3 genotypes including only one to three isolates were likely of external source. In contrast, numerous genotypes identified in the Senegalese population and belonging to either ST1 or ST2 were represented by only one isolate, implying a possible concurrent exposure of the villagers to different sources of infection or few sources containing several genotypes. These potential sources could therefore be multiple and particular to some villages as described for instance in Ndiol Maure and Malla Tack. In Ndiol Maure, seven of the 11 ST1 genotypes were represented by a single isolate and were only detected in this village. Similarly, among the 12 ST2 genotypes reported in Malla Tack, seven of them including only one isolate were uniquely found in that village. Consequently, even if part of the ST1 and ST2 infections occurs through human-to-human transmission in our Senegalese cohort, a large proportion would be the result of sporadic contamination from different well-located sources that remain to be determined. Future studies should be conducted to detect the presence of the parasite in animal and environmental samples collected from the corresponding villages.

## 5. Conclusions

The present survey completes the still insufficient data available for the African continent regarding *Blastocystis* sp.. The prevalence observed in our Senegalese cohort is extremely high as it exceeds 80%. Such prevalence is most probably linked to widespread and active transmission of the parasite due to precarious sanitary and hygiene conditions within the population and exposure to various contaminated environmental sources. Interestingly, the analysis of the intra-ST genetic diversity of the three main STs identified herein strongly suggests that the predominant ST3 is mainly transmitted by the inter-human route, whereas several sources of transmission occur for ST1 and ST2. Consequently, *Blastocystis* sp. may be a good sentinel for the detection of water contamination. It is also shown that the isolates considered with certainty to be of animal origin (ST7, ST10, and ST14) are in rather limited numbers in Senegal, highlighting the low risk of zoonotic transmission in this country as more globally in Africa. Finally, this large-scale field survey also provides valuable information to the local health authorities to implement urgent prevention and control measures in order to significantly reduce the incidence of *Blastocystis* sp. in this part of the world.

## Figures and Tables

**Table 1:** Water supplies and usage in the 10 Senegalese villages studied.

Villages	Water Sources and Usage
**Area of Saint-Louis**	
Maka Diama	Tap water ^a^ (Drinking, washing, domestic activities ^b^)
	Senegal River (Domestic activities ^b^, bathing, irrigating crops)
Ndiol Maure	Tap water ^a^ (Drinking, washing, domestic activities ^b^)
	Lampsar River (Domestic activities ^b^, bathing, irrigating crops)
Lampsar	Tap water ^a^ (Drinking, washing, domestic activities ^b^)
	Lampsar River (Domestic activities ^b^, bathing, irrigating crops)
Mbakhana	Tap water ^a^ (Drinking, washing, domestic activities ^b^)
	Lampsar River (Domestic activities ^b^, bathing, irrigating crops)
Ndiawdoune	Tap water ^a^ (Drinking, washing, domestic activities ^b^)
	Ngalam River (Domestic activities ^b^, bathing, irrigating crops)
**Area of Lake Guiers**	
Mbane	Tap water ^a^ (Drinking, washing, domestic activities ^b^)
	Lake Guiers (Domestic activities ^b^, bathing, irrigating crops)
Diokhor Tack	Lake Guiers (Drinking, washing, domestic activities ^b^, bathing, irrigating crops)
Foss	Lake Guiers (Drinking, washing, domestic activities ^b^, bathing, irrigating crops)
	Public and private water wells (Drinking, washing)
Malla	Tap water non-potable at a single point in the village (Drinking, washing, domestic activities ^b^)
	Lake Guiers (Drinking, washing, domestic activities ^b^, bathing, irrigating crops)
Malla Tack	Tap water non-potable in 3 houses of the village (Washing, domestic activities ^b^)
	Lake Guiers (Drinking, washing, domestic activities ^b^, bathing, irrigating crops)

^a^ Tap water in less than 20% of the dwellings (most prosperous part of the population). ^b^ Domestic activities consisting mainly of cooking, household cleaning, dishes and laundry.

**Table 2:** Prevalence and ST distribution of *Blastocystis* sp. in the 10 Senegalese villages screened in the present study.

Villages	Samples (*n*)	Positive Samples (*n*)	Prevalence (%)	*Blastocystis* sp. STs						
				ST1	ST2	ST3	ST7	ST10	ST14	MI ^a^
**Area of Saint-Louis**										
Maka Diama	75	57	76.0	15	15	13	0	1	0	13
Ndiol Maure	71	47	66.2	18	12	9	0	0	0	8
Lampsar	86	77	89.5	8	27	27	2	0	0	13
Mbakhana	81	72	88.9	11	27	13	0	0	1	20
Ndiawdoune	50	50	100	0	24	10	1	0	1	14
Total area	363	303	83.5	52	105	72	3	1	2	68
**Area of the Lake Guiers**										
Mbane	89	46	51.7	17	11	10	0	0	0	8
Diokhor Tack	104	80	76.9	22	32	7	0	0	0	19
Foss	25	23	92.0	7	8	3	0	0	0	5
Malla	54	51	94.4	2	34	3	0	0	0	12
Malla Tack	96	85	88.5	13	36	12	0	1	0	23
Total area	368	285	77.4	61	121	35	0	1	0	67
**Grand total**	**731**	**588**	**80.4**	**113**	**226**	**107**	**3**	**2**	**2**	**135**

^a^ MI, Mixed infections.

**Table 3:** Distribution of ST1, ST2, and ST3 genotypes of *Blastocystis* sp. in the 10 Senegalese villages.

Villages	ST1 Isolates	ST1 Genotypes/15	ST2 Isolates	ST2 Genotypes/20	ST3 Isolates	ST3 Genotypes/8	Total ST1, ST2 and ST3 Isolates	Total ST1, ST2 and ST3 Genotypes/43	Total Solates/Total Genotypes
**Area of Saint-Louis**									
Maka Diama	15	3	15	5	13	4	43	12	3.6
Ndiol Maure	18	11	12	6	9	4	39	21	1.9
Lampsar	8	2	27	3	27	4	62	9	6.9
Mbakhana	11	4	27	4	13	5	51	13	3.9
Ndiawdoune	0	0	24	2	10	2	34	4	8.5
Average							229	30	7.6
**Area of Lake Guiers**									
Mbane	17	6	11	4	10	3	38	13	2.9
Diokhor Tack	22	4	32	4	7	1	61	9	6.8
Foss	7	5	8	3	3	3	18	11	1.6
Malla	2	1	34	3	3	2	39	6	6.5
Malla Tack	13	6	36	12	12	4	61	22	2.8
Average							217	27	8.0

**Table 4:** Prevalence and ST distribution of *Blastocystis* sp. in African countries.

African Region/Countries	Prevalence	Subtyped Isolates	Subtyping Method	*Blastocystis* sp. STs									Mixed Infection	Reference
				ST1	ST2	ST3	ST4	ST5	ST6	ST7	ST10	ST14		
**North Africa**														
Tunisia	NA ^a^	61	Sequencing	18	10	31	1	0	0	1	0	0	0	[47]
Libya	28.0%	38	Sequencing	19	3	15	0	0	0	1	0	0	0	[31]
Libya	NA ^a^	48	Sequencing	26	13	9	0	0	0	0	0	0	0	[48]
Egypt	NA ^a^	36	PCR-STS ^b^	6	0	30	0	0	0	0	0	0	0	[49]
Egypt	NA ^a^	110	PCR-STS ^b^	15	0	49	0	0	33	13	0	0	0	[50]
Egypt	NA ^a^	33	Sequencing	0	0	33	0	0	0	0	0	0	0	[51]
Egypt	NA ^a^	21	Sequencing	4	4	13	0	0	0	0	0	0	0	[36]
Egypt	NA ^a^	44	PCR-STS ^b^	8	0	24	0	0	8	4	0	0	0	[52]
Total		391		96	30	204	1	0	41	19	0	0	0	
**West Africa**														
Nigeria	84.0%	127	Sequencing	51	42	33	0	0	0	1	0	0	0	[37]
Nigeria	49.0%	22	Sequencing	10	0	9	3	0	0	0	0	0	1	[31]
Côte d’Ivoire	58.2%	64	Sequencing	32	14	18	0	0	0	0	0	0	0	[38]
Côte d’Ivoire	70.0%	0	NA ^a^	0	0	0	0	0	0	0	0	0	0	[45]
Liberia	70.0%	25	Sequencing	7	7	8	3	0	0	0	0	0	5	[31]
Senegal	80.4%	453	Sequencing	113	226	107	0	0	0	3	2	2	135	Present study
Senegal	100%	103	Sequencing	29	21	51	2	0	0	0	0	0	0	[12]
Mali	49.7%	0	NA ^a^	0	0	0	0	0	0	0	0	0	0	[46]
Total		794		242	310	226	8	0	0	4	2	2	141	
**Central Africa**														
Angola	25.6%	75	Sequencing	23	23	27	0	1	0	1	0	0	0	[42]
Cameroon	88.2%	65	Sequencing	23	9	33	0	0	0	0	0	0	0	[13]
Total		140		46	32	60	0	1	0	1	0	0	0	
**East Africa**														
Tanzania	81.8%	34	Metagenomics	11	13	10	0	0	0	0	0	0	0	[13]
Tanzania	55.6%	15	Metagenomics	1	12	2	0	0	0	0	0	0	0	[43]
Tanzania	60.9%	92	Sequencing	36	28	27	0	0	0	1	0	0	0	[41]
Tanzania	NA ^a^	6	Sequencing	1	3	2	0	0	0	0	0	0	0	[53]
Total		147		19	56	41	0	0	0	1	0	0	0	
**Southern Africa**														
Madagascar	64.5%	158	Sequencing	80	36	42	0	0	0	0	0	0	13	[44]
Total		158		80	36	42	0	0	0	0	0	0	13	
Grand total		1630		513	464	573	9	1	41	25	2	2	154	

^a^ NA, not applicable. ^b^ STS, subtype-specific sequence-tagged site.
